# Fowl adenovirus serotype 4 enters leghorn male hepatocellular cells via the clathrin-mediated endocytosis pathway

**DOI:** 10.1186/s13567-023-01155-z

**Published:** 2023-03-14

**Authors:** Ting Wang, Lizhen Wang, Wei Li, Xiaolan Hou, Wenchi Chang, Bo Wen, Shuizhong Han, Yan Chen, Xuefeng Qi, Jingyu Wang

**Affiliations:** College of Veterinary Medicine, Northwest A&F University, 712100 Yangling, Shaanxi China

**Keywords:** FAdV-4, entry, LMH cells, clathrin, endocytosis

## Abstract

Hepatitis-hydropericardium syndrome (HHS) induced by fowl adenovirus serotype-4 (FAdV-4) has caused large economic losses to the world poultry industry in recent years. HHS is characterized by pericardial effusion and hepatitis, manifesting as a swollen liver with focal necroses and petechial haemorrhage. However, the process of FAdV-4 entry into hepatic cells remains largely unknown. In this paper, we present a comprehensive study on the entry mechanism of FAdV-4 into leghorn male hepatocellular (LMH) cells. We first observed that FAdV-4 internalization was inhibited by chlorpromazine and clathrin heavy chain (CHC) knockdown, suggesting that FAdV-4 entry into LMH cells depended on clathrin. By using the inhibitor dynasore, we showed that dynamin was required for FAdV-4 entry. In addition, we found that FAdV-4 entry was dependent on membrane cholesterol, while neither the knockdown of caveolin nor the inhibition of a tyrosine kinase-based signalling cascade affected FAdV-4 infection. These results suggested that FAdV-4 entry required cholesterol but not caveolae. We also found that macropinocytosis played a role, and phosphatidylinositol 3-kinase (PI3K) was required for FAdV-4 internalization. However, inhibitors of endosomal acidification did not prevent FAdV-4 entry. Taken together, our findings demonstrate that FAdV-4 enters LMH cells through dynamin- and cholesterol-dependent clathrin-mediated endocytosis, accompanied by the involvement of macropinocytosis requiring PI3K. Our work potentially provides insight into the entry mechanisms of other avian adenoviruses.

## Introduction

Fowl adenoviruses (FAdV) belong to the family *Adenoviridae*, genus *Aviadenovirus* and are further clustered into five species (FAdV-A-E) with 12 serotypes (FAdV-1-8a, 8b-11) based on analyses-based restriction enzyme digestion and serum cross-neutralization assays [1]. Notably, infection with different serotypes of FAdV leads to different clinical symptoms. Inclusion body hepatitis (IBH) is related mainly to infection with FAdV-8a, FAdV-8b, FAdV-2, and FAdV-11 [2, 3]; gizzard erosion (GE) is generally induced by infection with FAdV-1 [4]; and hepatitis-hydropericardium syndrome (HHS) is predominantly caused by FAdV-4 [5]. In 1987, HHS was first reported in Pakistan and subsequently spread to many countries in Asia, America, and Europe, causing significant economic losses in the poultry industry worldwide [6, 7].

FAdV-4 carries a genome of linear double-stranded DNA consisting of 45 kb, encoding 11 structural proteins and 32 nonstructural proteins. The exposed structural proteins, hexon, fibre, and penton base, constitute the viral capsid [8]. In adenoviral infection, the fibre knob is the primary attachment site for the cellular receptor coxsackievirus and adenovirus receptor (CAR), while the penton base is involved in secondary interactions required for viral entry into cells [9]. In contrast to other FAdV serotypes, the FAdV-1, FAdV-4, and FAdV-10 serotypes carry two fibres (fibre-1 and fibre-2) inserted in each penton base. Recent research has shown that fibre-1 is critical for binding CAR [10] and directly mediates infection with FAdV-4 through its shaft and knob domains [11], whereas fibre-2 and hexon are closely associated with the virulence of FadV-4 [12]. However, the steps following the initial attachment of FadV-4 to the host cell receptor remain poorly characterized.

To date, many animal viruses have evolved to exploit various endocytic pathways for entry, including clathrin-mediated endocytosis, macropinocytosis, caveolae-mediated endocytosis, and clathrin- and caveolae-independent endocytosis [13–17]. Most of the studies carried out on adenovirus endocytosis have been performed with human adenovirus type (HAdV) [14, 18–22]. In the case of HAdV-2 and HAdV-5 infection of epithelial cells, viruses bind to the primary receptor CAR, and then, CAR-docked particles activate integrin coreceptors, triggering a variety of cellular responses, including clathrin-mediated endocytosis, which involves the large GTPase dynamin and adaptor protein 2 [18]. Moreover, macropinocytosis is an accessory pathway triggered by HAdV2 in respiratory epithelial cells [19]. For other adenoviruses, such as HAdV-3, the viruses primarily induce macropinocytosis for entry and infection [20]. Additionally, viral entry pathways are cell-type specific. For example, the epidemic keratoconjunctivitis (EKC) agent HAdV-37 enters human corneal epithelial cells via clathrin-dependent endocytosis but enters human keratocytes through caveolae in a caveolin 1–dependent manner [21, 22]. Previously, we explored the entry pathway of egg drop syndrome virus (EDSV), a member of the Aviadenovirus family, and demonstrated that EDSV utilized clathrin-mediated endocytosis to enter duck embryonic fibroblast (DEF) cells [23]. Given these reports, it is obvious that adenovirus entry is a complex process wherein different virus strains may leverage different mechanisms to enter different cell types.

In a previous study, we preliminarily elucidated the kinetics of FAdV-4 internalization into leghorn male hepatocellular (LMH) cells by analysing the uptake of the virus into cells at indicated time points using confocal laser scanning microscopy (CLSM) and transmission electron microscopy (TEM) [24]. The present study aimed to further investigate the internalization mechanism of FAdV-4 in LMH cells. We used chemical inhibitors and RNA interference (RNAi) silencing to examine the cellular molecules involved in the FAdV-4 entry process. The results indicate FAdV-4 enters LMH cells via a dynamin- and cholesterol-dependent, clathrin-mediated endocytic pathway and involves macropinocytosis and PI3K, independent of caveolae.

## Materials and methods

### Cell lines and viruses

Leghorn male hepatocellular cells (LMH cells), kindly provided by Prof. Yunfeng Wang (Harbin Veterinary Research Institute, Heilongjiang, China), were cultured in Dulbecco’s modified Eagle’s medium (Gibco, Grand Island, NY, USA) supplemented with 10% foetal bovine serum (Gibco). The FAdV-4 strain SX17 (GenBank: MF592716.1) was isolated and maintained in our laboratory.

### Inhibitors, antibodies, shRNAs, and plasmids

Ammonium chloride (NH_4_Cl) and chloroquine were purchased from Sigma (St. Louis, MO, USA). Chlorpromazine (CPZ), methyl-β-cyclodextrin (MβCD), dynasore, and wortmannin were purchased from TargetMol (Boston, MA, USA). 5-Ethyl-N-isopropyl amiloride (EIPA) was purchased from MedChemExpress (Monmouth Junction, NJ, USA).

An anti-FAdV-4-fiber2 monoclonal antibody (mAb 3C2) was kindly provided by Prof. Jianqiang Ye (Yangzhou University, Yangzhou, Jiangsu, China). A rabbit polyclonal anti-FAdV-4-penton antibody was generated by our laboratory. Rabbit polyclonal anti-caveolin-1 and clathrin HC antibodies were purchased from Santa Cruz Biotechnology (CA, USA). Anti-β-actin antibodies, HRP-conjugated secondary antibodies, and FITC-conjugated anti-mouse and anti-rabbit IgG were purchased from TransGen Biotech (Beijing, China). TRITC-phalloidin and FITC-dextran were purchased from Sigma.

A pGFP-V-RS (vector), scramble shRNAs, and shRNAs targeting clathrin heavy chain (CHC) (shCHC) were kindly provided by Dr Hung-Jen Liu (Institute of Molecular Biology, National Chung Hsing University, Taichung, Taiwan). siRNA targeting caveolin-1 (5′-CCACTTTCACTGTAACAAA-3′) and negative siRNA were synthesized by RiboBio (Guangzhou, China).

### Cell viability assay

Cell viability was measured using a commercial CCK-8 kit (TargetMol) according to the manufacturer’s instructions. Briefly, LMH cells were seeded in 96-well cell culture plates. After incubation for 24 h, serial concentrations of pharmacological inhibitors were added in triplicate and incubated for 12 h, and 10 µL of CCK-8 reagent was added and incubated for an additional 2 h. The optical density was read at 450 nm.

### Chemical inhibitor treatment and virus infection

Monolayers of LMH cells grown in 24-well plates were pretreated with different inhibitors at the indicated concentrations for 1 h at 37 °C. Noncytotoxic concentrations for all inhibitors were determined by serial dilution on LMH cells, and cytotoxicity was quantified via a cell viability assay, as described above. Cells were then inoculated with FAdV-4 at a multiplicity of infection (MOI) of 20 at 4 °C to allow for viral attachment. After adsorption for 1 h, unbound viruses were removed by washing twice with phosphate-buffered saline (PBS). Then, the cells were cultured in the presence of drugs in DMEM–2% FBS at 37 °C. After one hour of incubation, cells were washed with PBS and treated with proteinase K (2 mg/mL) (Solarbio, Beijing, China) for 30 min at 4 °C to remove adsorbed but not internalized viruses. Proteinase K was then inactivated, and the cells were washed three times with PBS–0.4% BSA. A cholesterol supplementation assay was performed to further examine the role of cholesterol in FAdV-4 entry. Cells were treated with MβCD (10 mM) for 1 h at 37 °C, followed by the addition of 50 µg/mL cholesterol (Sigma) at different time points before, during, and after infection with FAdV-4. At the indicated time points, cells were lysed by three freeze‒thaw cycles and collected for DNA isolation. Viral DNA was prepared using an EasyPure DNA kit (TIANGEN Biotech, Beijing, China), and quantitative real-time PCR (qPCR) was performed as previously described [24]. qPCR was conducted with specific primers for a fragment of the hexon gene (KU569296.1), and the primers were designed as follows: F: 5′-ACAGG TCCTCAGCTACAAGA-3′ and R: 5′-TGACCCTAACGGTGTCGA-3′. Moreover, cells were collected for Western blot analysis and prepared for confocal laser scanning microscopy (CLSM) analysis.

### Transfection and gene silencing

LMH cells grown to 75% confluence in 12-well cell culture plates were transfected with 2 µg/well CHC shRNA plasmids or 100 nM caveolin-1 siRNA. Transfection was performed according to the protocol of the manufacturer of the TurboFect transfection reagent (Thermo Fisher, Waltham, MA, USA). At 48 h post-transfection, cells were infected with FAdV-4 at an MOI of 20 and then processed as described above. At the indicated time points, cells were collected for qPCR and Western blot analysis. Silencing efficiency was quantified by Western blot analysis.

### Confocal laser scanning microscopy

LMH cells were grown on coverslips in 24-well plates, infected with FAdV-4 at an MOI of 20 at 4 °C and incubated for 1 h, and then, the cells were incubated to 37 °C. At the indicated times, cells were fixed with 4% paraformaldehyde, permeabilized with 0.5% Triton X-100, blocked with 3% bovine serum albumin (BSA), and incubated with anti-FAdV-4-fiber2 monoclonal antibodies at 4 °C overnight. Then, the cells were incubated with FITC-conjugated secondary antibodies. FITC-dextran (1 mg/mL; Sigma) was used as a control for EIPA. LMH cells were incubated with an inhibitor for 1 h at 37 °C, after which a marker was added. Cell incubation was continued for 30 min, and the cells were then processed as described above. Actin filaments were stained with TRITC-phalloidin (2 mg/mL) for 60 min at 25 °C. Finally, the cells were treated with 1 mg/mL DAPI solution for 10 min and analysed by confocal microscopy (CLSM Leica SP8, Germany).

### Western blot assay

Whole-cell lysates were generated by adding 5×SDS‒PAGE sample buffer to cells. Samples were boiled for 5 min and separated by SDS‒PAGE. Proteins were then transferred to polyvinylidene difluoride (PVDF) membranes (Millipore, Billerica, MA, USA). The membranes were blocked with 5% nonfat milk and incubated with primary antibodies, followed by horseradish peroxidase (HRP)-conjugated secondary antibodies. Bound antibodies were detected with ECL immunoblot detection reagent (Millipore).

### Statistical analysis

All data are presented as the mean ± standard deviation (SD) on the basis of at least three independent experiments. Comparisons between groups were performed using a two-tailed Student’s *t* test with GraphPad Prism 6.0 software (GraphPad Software, San Diego, CA, USA). Statistical significance: *, *P* < 0.05, **, *P* < 0.01 and ***, *P* < 0.001.

## Results

### FAdV-4 enters LMH cells via the clathrin-mediated endocytosis pathway

**Figure 1:**
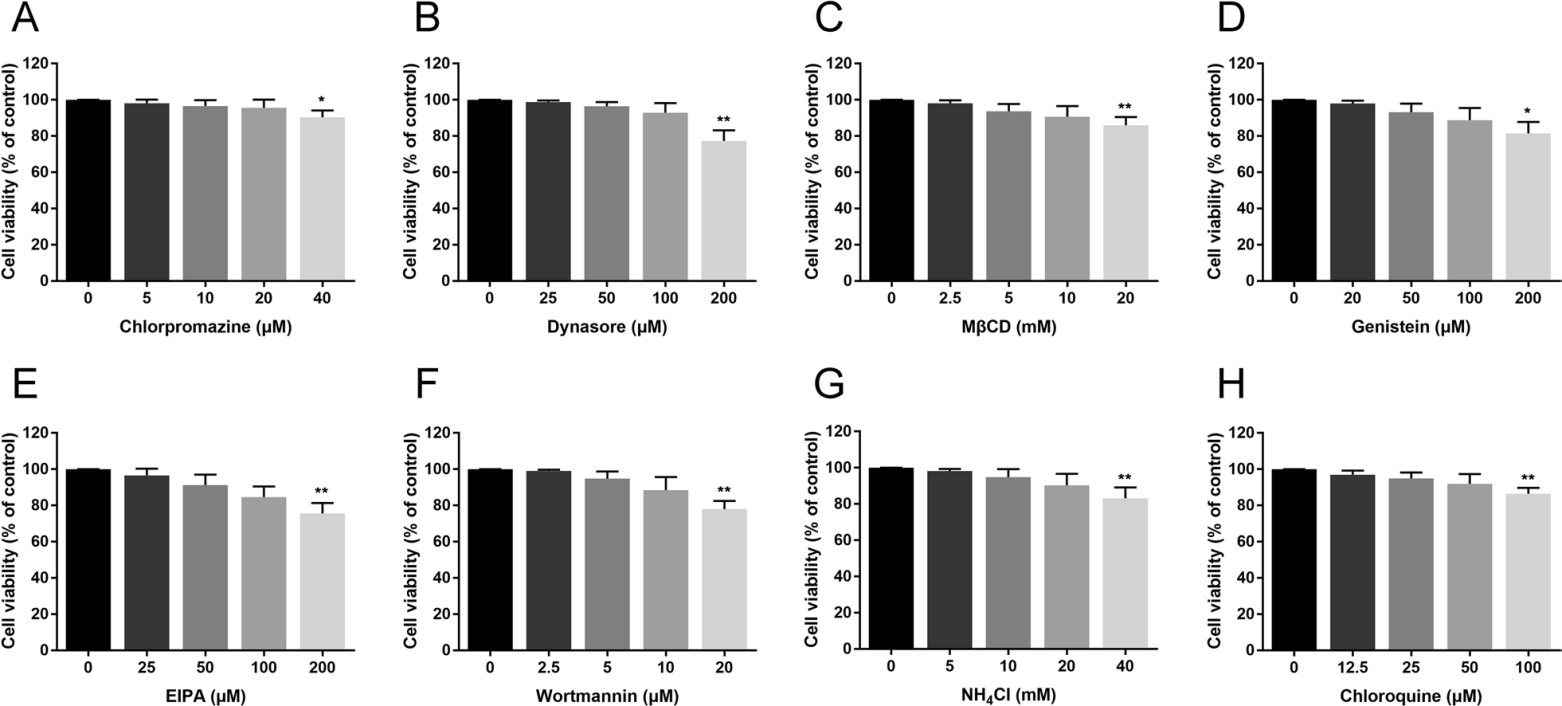
**Cell viability assays determined the cytotoxicity of inhibitors in LMH cells. A**–**H** All cells treated with inhibitors were subjected to viability assays to evaluate their cytotoxicity of the inhibitors to LMH cells. Cells were exposed to the indicated concentrations of inhibitors for 12 h. ^∗^, *P* < 0.05; ^∗∗^, *P* < 0.01.

**Figure 2:**
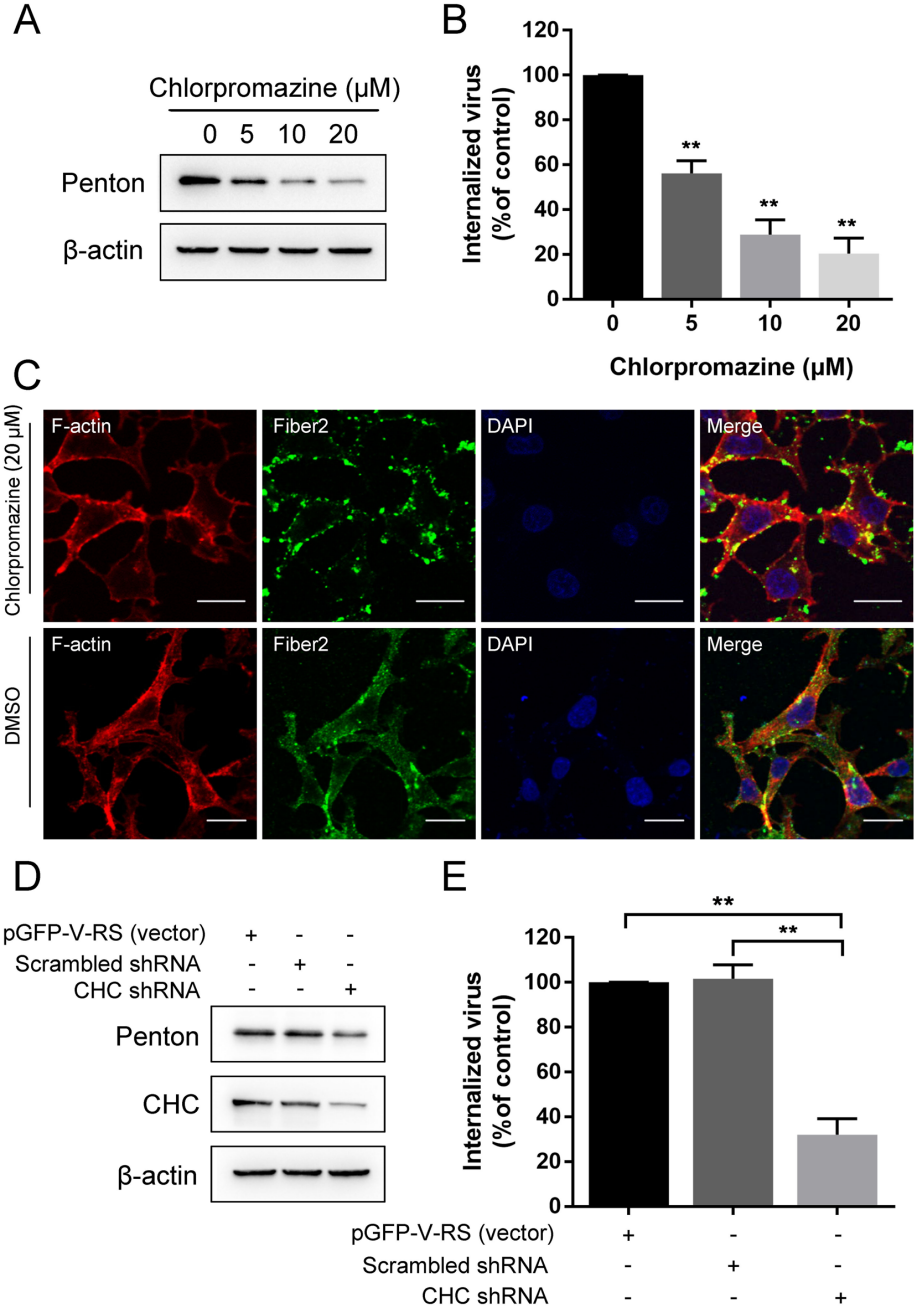
**FAdV-4 entry into LMH cells depends on clathrin-mediated endocytosis. A** Western blot analysis after the entry of FAdV-4 into chlorpromazine-treated cells. β-Actin was used as the internal control. **B** qPCR analysis after the entry of FAdV-4 into chlorpromazine-treated cells. **C** CLSM analysis of F-actin (red), anti-FAdV-4 fibre 2 (green) and DAPI (blue) in FAdV-4-infected LMH cells pretreated with chlorpromazine. **D** Western blot analysis of the protein expression of CHC after the entry of FAdV-4 in cells transfected with CHC shRNA and mock control vectors. **E** qPCR analysis after the entry of FAdV-4 into cells transfected with CHC shRNA and mock control vectors. Bars, 20 μm. *, *P* < 0.05; **, *P* < 0.01.

It has been established that adenovirus enters many cell types via canonical clathrin-mediated endocytosis [18]. To confirm whether FAdV-4 enters LMH cells via clathrin-mediated endocytosis, chlorpromazine (CPZ) was used to specifically block the clathrin-mediated endocytic pathway because it prevents the assembly of clathrin-coated pits (CCPs) [25]. Possible drug-induced cytotoxic effects were assessed by CCK-8 cell viability assay. As shown in Figure 1A, cells tolerated chlorpromazine concentrations as high as 20 µM. LMH cells were pretreated with various concentrations of chlorpromazine, and then, the cells were infected with FAdV-4. The effect of chlorpromazine on FAdV-4 internalization was assessed by measurements of the amount of penton protein that was expressed as well as the viral DNA copy numbers. Western blot analysis showed that chlorpromazine treatment before FAdV-4 infection significantly inhibited virus internalization in a dose-dependent manner (Figure 2A). Additionally, pretreatment with 20 µM chlorpromazine resulted in a 79.6% reduction in the viral DNA copy number (Figure 2B). Moreover, a staining assay revealed the fluorescence signals of FAdV fiber2 on the membrane of cells pretreated with 20 µM chlorpromazine (Figure 2C).

To confirm these data, we used shRNA to specifically knock down the expression of clathrin heavy chain (CHC), which is a key component for regulating the formation and disassembly of the clathrin lattice. At 48 h post-transfection, FAdV-4 internalization assays were performed, and we found that transfection of CHC shRNA specifically decreased the expression of CHC in FAdV-4-infected cells (Figure 2D). Additionally, a marked reduction in the penton protein level and viral DNA copy number in the presence of CHC shRNA was observed compared to that in negative controls (Figures 2D and E). Taken together, these results strongly suggest that FAdV-4 entered LMH cells via canonical clathrin-mediated endocytosis.

### FAdV-4 endocytosis into LMH cells depends on dynamin

**Figure 3:**
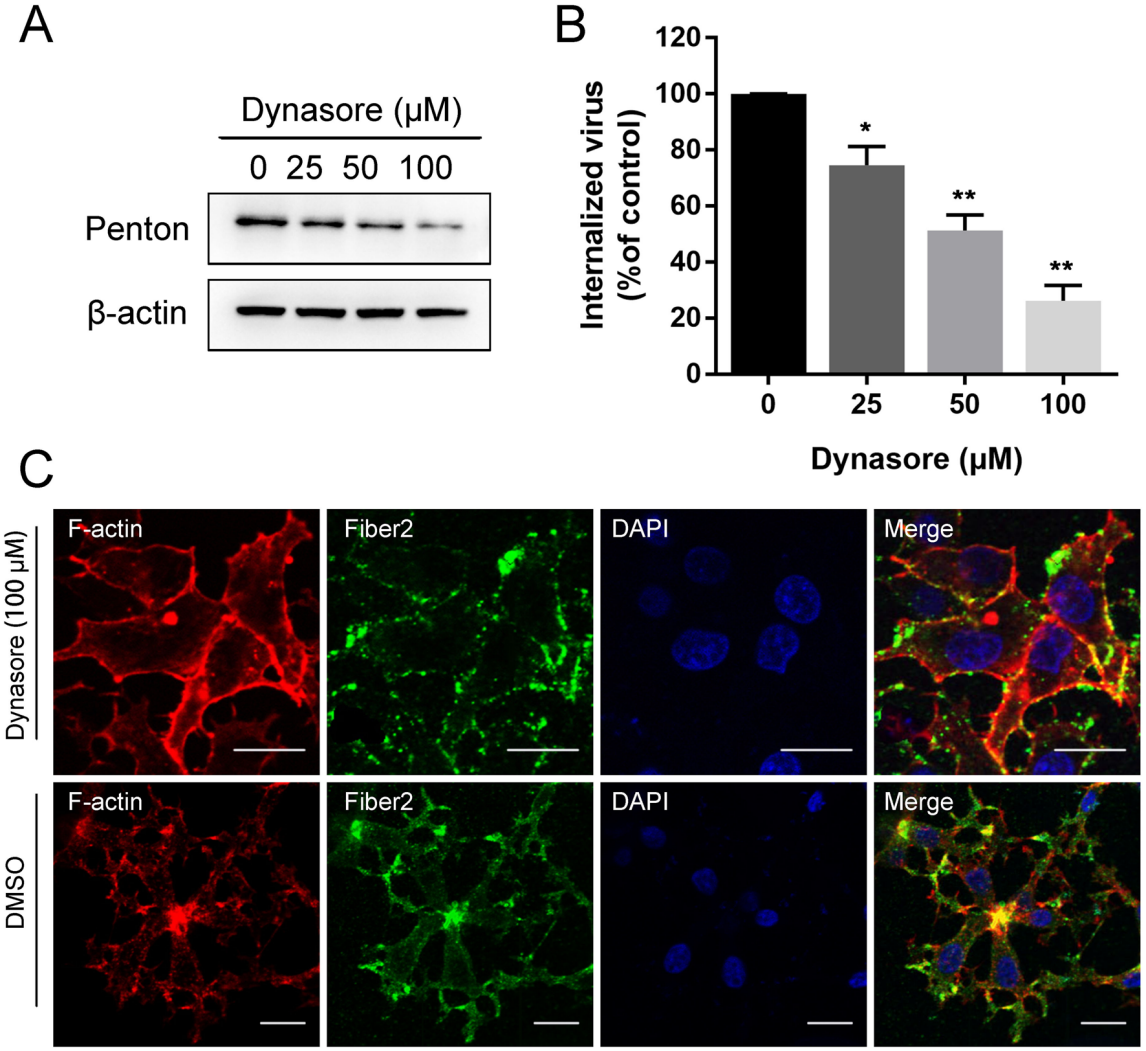
**
FAdV-4 entry depends on dynamin. A** Western blot analysis after the entry of FAdV-4 into dynasore-treated cells. β-Actin was used as the internal control. **B** qPCR analysis after the entry of FAdV-4 into dynasore-treated cells. **C** CLSM analysis of F-actin (red), anti-FAdV-4 fibre 2 (green) and DAPI (blue) in FAdV-4-infected LMH cells pretreated with dynasore. Bars, 20 μm. *, *P* < 0.05; **, *P* < 0.01.

Dynamin, a GTPase that is required for the formation of vesicles, plays an essential role in clathrin- and caveolae-mediated endocytosis [26]. Since dynamin has been reported to be involved in Ad2/Ad5 endocytosis [27], we analysed its role in FAdV-4 internalization. LMH cells were pretreated with dynasore, a cell-permeable, noncompetitive dynamin GTPase activity inhibitor, and infected the cells with FAdV-4. No cytotoxicity was observed in the cells treated with dynasore at concentrations as high as 100 µM (Figure 1B). As shown in Figure 3A, FAdV-4 internalization was significantly inhibited by dynasore pretreatment in a dose-dependent manner. In the presence of dynasore, the DNA copy number of FAdV-4 was decreased in a dose-dependent manner. FAdV-4 entry was inhibited by more than 70% when treated with 100 µM dynasore (Figure 3B). Similarly, a more intense green fluorescence signal specific to FAdV-4 fiber2 was observed on the membrane of 100 µM dynasore-treated cells compared with control cells (Figure 3C). Taken together, the results of dynasore pretreatment suggest that dynamin is required for FAdV-4 entry.

### Plasma membrane cholesterol is required for FAdV-4 infection

**Figure 4:**
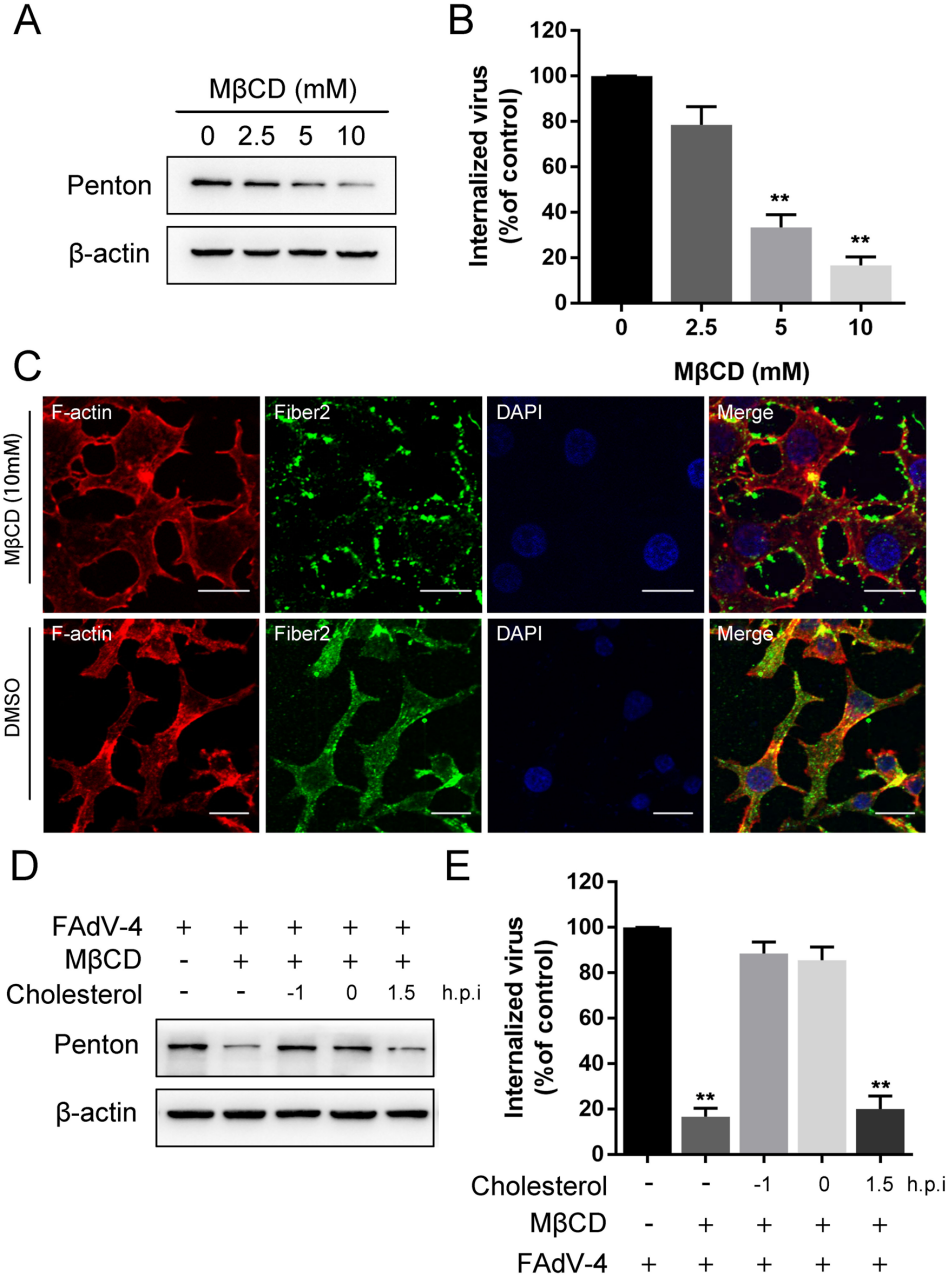
**FAdV-4 entry into LMH cells requires plasma membrane cholesterol. A **Western blot analysis after the entry of FAdV-4 into MβCD-treated cells. β-Actin was used as the internal control. **B** qPCR analysis after the entry of FAdV-4 into MβCD-treated cells. **C** CLSM analysis of F-actin (red), anti-FAdV-4 fibre 2 (green) and DAPI (blue) in FAdV-4-infected LMH cells pretreated with MβCD. Bars, 20 μm. *, *P* < 0.05; **, *P* < 0.01.

Membrane cholesterol is a prominent component of lipid rafts and necessary for membrane ruffling and actin reorganization [28]. Increasing evidence suggests that cholesterol-rich membrane rafts are involved in the entry of many viruses into cells [29, 30]. In this study, we confirmed the role of cholesterol in FAdV-4 entry through a series of experiments. We used MβCD to deplete cholesterol in LMH cells to disrupt lipid rafts. MβCD treatment at 10 mM did not affect cell viability (Figure 1C). The effects of MβCD on FAdV-4 entry were determined by viral internalization assay. When LMH cells were pretreated with increasing concentrations of MβCD before infection, the levels of penton protein were reduced in a dose-dependent manner (Figure 4A). Consistent with the Western blot results, qPCR further showed that the FAdV-4 DNA copy number in treated cells was significantly reduced in a dose-dependent manner (Figure 4B). A concentration of 10 mM MβCD led to a greater than 80% reduction in FAdV-4 infection compared to that induced by DMSO (Figure 4B). Moreover, indirect immunofluorescence demonstrated that the treatment of the cells with 10 mM MβCD induced the expression of green fluorescence signals specific to the FAdV-4 fiber2 proteins on the cell membrane and that this expression was not observed in control cells (Figure 4C). To confirm the role of cholesterol, cells were treated with MβCD (10 mM) for 1 h at 37 °C, followed by treatment with 50 µg/mL cholesterol at different time points before, during, and after infection with FAdV-4. The entry of FAdV-4 was reversed by cholesterol supplementation before or during the internalization period, as demonstrated by the level of penton protein and viral DNA copy number (Figures 4D and E); however, little effect was observed when cholesterol was added at the post-entry stage. These results reveal that plasma membrane cholesterol is important to the early steps of FAdV-4 infection.

### Caveolae are not required for FAdV-4 entry into LMH cells

**Figure 5:**
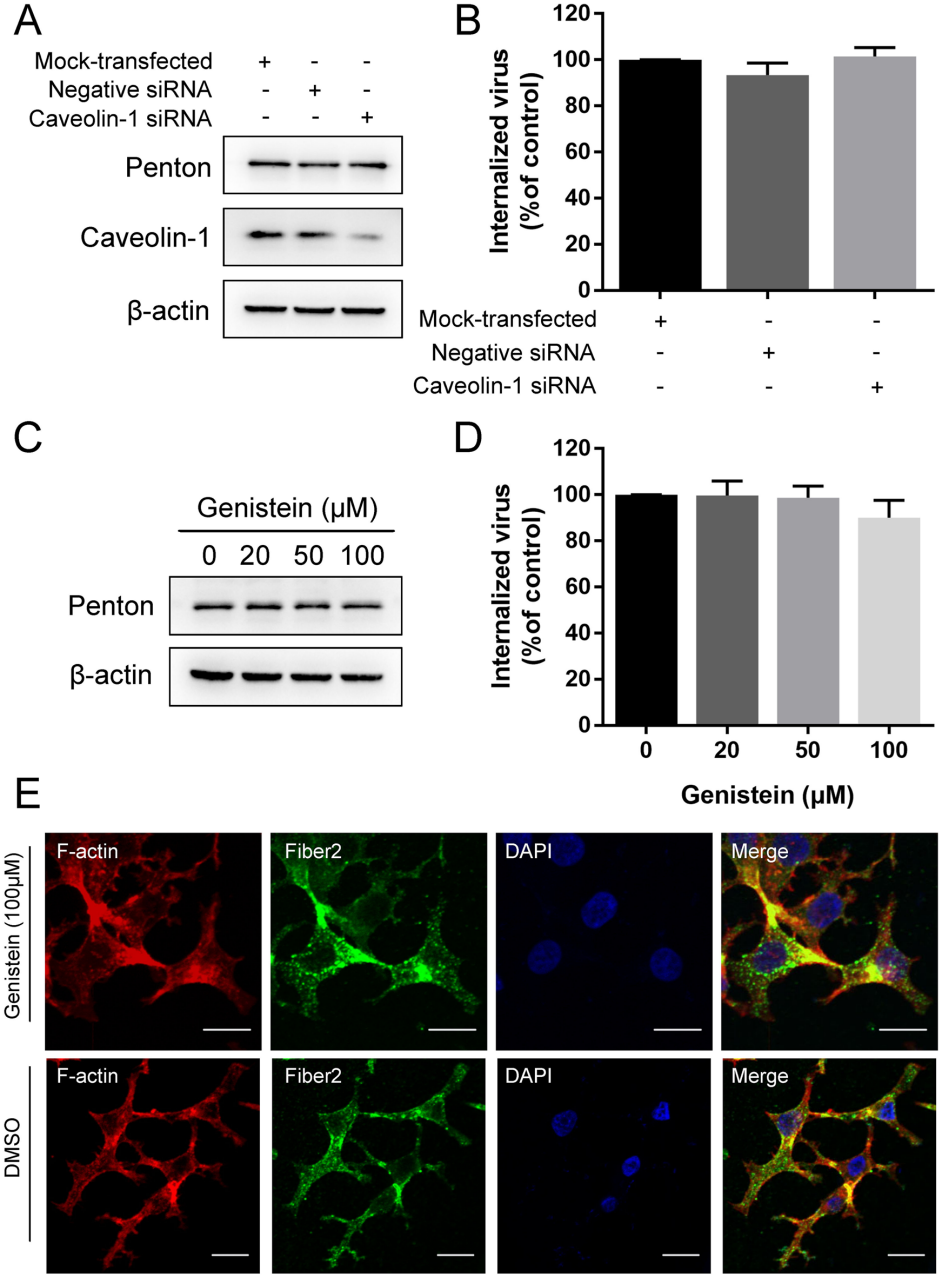
**Caveolae-mediated endocytosis is not the pathway by which FAdV-4 enters LMH cells. A** Western blot analysis of the protein expression of caveolin-1 after the entry of FAdV-4 in cells transfected with caveolin-1 siRNA and Neg.siRNA. **B** qPCR analysis after the entry of FAdV-4 into cells transfected with caveolin-1 siRNA and Neg.siRNA. **C** Western blot analysis after the entry of FAdV-4 into genistein-treated cells. β-Actin was used as the internal control. **D** qPCR analysis after the entry of FAdV-4 into genistein-treated cells. **E** CLSM analysis of F-actin (red), anti-FAdV-4 fibre 2 (green) and DAPI (blue) in FAdV-4-infected LMH cells pretreated with genistein. Bars, 20 μm. *, *P* < 0.05; **, *P* < 0.01.

To determine whether FAdV-4 enters cells through multiple endocytic pathways, we examined the role of caveolae in FAdV-4 entry into LMH cells. Given that caveolin-1 is essential for the formation and stability of caveolae, we constructed a specific small-interfering RNA (siRNA) targeting caveolin-1 to investigate the effect of caveolae. The silencing efficiency of siRNA-mediated knockdown of caveolin-1 in LMH cells was analysed by Western blot analysis (Figure 5A), followed by a FAdV-4 internalization assay. However, the protein level of penton in caveolin-1 siRNA-transfected cells was similar to that cells that were not transfected with siRNA (Figure 5A). The qPCR analysis data also supported the finding that downregulation of caveolin-1 expression did not significantly influence FAdV-4 entry (Figure 5B). These results indicate that caveolin-1 is not involved in FAdV-4 infection in LMH cells.

The caveolae-mediated endocytosis pathway requires the activation of a tyrosine kinase-based signalling cascade and initiates a series of additional signal transduction pathways [31]. Therefore, we used an established tyrosine kinase inhibitor, genistein, to ascertain whether FAdV-4 enters LMH cells through caveolae-mediated endocytosis. A cell viability analysis was conducted, and a genistein working concentration range of 20–100 µM was established (Figure 1D). Not surprisingly, treating cells with increasing doses of genistein failed to inhibit FAdV-4 internalization. No significant differences in the level of penton protein and number of viral DNA copies were observed between genistein-treated and DMSO-treated groups (Figures 5 C and D). Moreover, genistein treatment did not induce the expression of a green fluorescence specific to FAdV-4 fiber2 proteins on the cell membrane, in contrast to its effect on control cells (Figure 5E). These findings provide evidence that the caveolae-mediated endocytosis pathway is not involved in FAdV-4 internalization.

### FAdV-4 entry depends on macropinocytosis and requires PI3K

**Figure 6:**
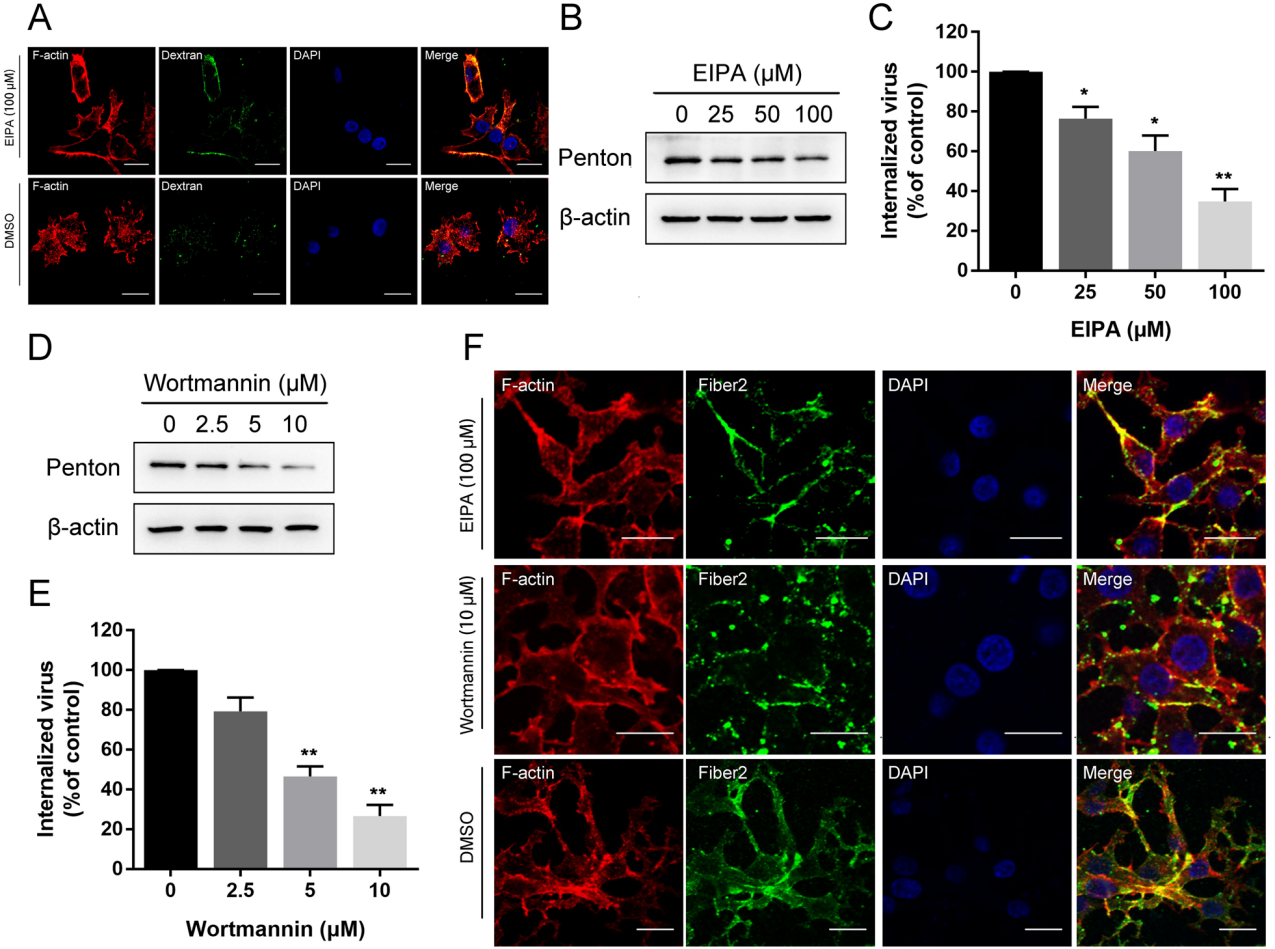
**FAdV-4 entry depends on macropinocytosis and requires PI3K. A** CLSM analysis of F-actin (red), FITC-dextran (green) and DAPI (blue) in FAdV-4-infected LMH cells pretreated with EIPA. **B** Western blot analysis after the entry of FAdV-4 into EIPA- or wortmannin-treated cells. β-Actin was used as the internal control. **B** qPCR analysis after the entry of FAdV-4 into EIPA- or wortmannin-treated cells. **C** CLSM analysis of F-actin (red), anti-FAdV-4 fibre 2 (green) and DAPI (blue) in FAdV-4-infected LMH cells pretreated with EIPA or wortmannin. Bars, 20 μm. *, *P* < 0.05; **, *P* < 0.01.

In addition to clathrin-dependent endocytic, macropinocytosis is an entry pathway for some adenoviruses [19, 32]. To examine the potential involvement of macropinocytosis in FAdV-4 entry into LMH cells, we examined the effect of 5-ethyl-N-isopropyl amiloride (EIPA), an inhibitor of macropinocytosis that blocks Na^+^/H^+^ exchange [33], on FAdV-4 internalization. EIPA treatment at 100 µM did not affect cell viability (Figure 1E). As shown in Figure 6A and 100 µM EIPA treatment caused significant induction of dextran uptake (a specific fluid phase marker of macropinocytosis). Importantly, EIPA treatment before viral infection significantly inhibited viral entry. When LMH cells were pretreated with increasing concentrations of EIPA before infection, the amount of penton protein and the number of viral DNA copies were significantly reduced in a dose-dependent manner (Figures 6B and C). A confocal microscopy assay confirmed that pretreatment with EIPA strongly inhibited FAdV-4 internalization (Figure 6F). These findings indicate that FAdV-4 entry involves Na^+^/H^+^ exchange. During macropinocytosis, phosphatidylinositol 3-kinase (PI3K) is required for ruffling and macropinosomes formation [34]. To further examine the role of PI3K in FAdV-4 entry, LMH cells were treated with wortmannin, a specific PI3K inhibitor. Wortmannin treatment at 10 µM did not affect cell viability (Figure 1F) but significantly inhibited the infection rate of FAdV-4, as evidenced by the penton protein level and number of viral DNA copies (Figure 6D, E). Furthermore, wortmannin treatment induced the emission of green fluorescence signals specific to the FAdV-4 fiber2 proteins on the cell membrane (Figure 6F). In summary, we conclude that FAdV-4 entry into LMH cells depends on macropinocytosis.

### FAdV-4 entry is pH independent

**Figure 7:**
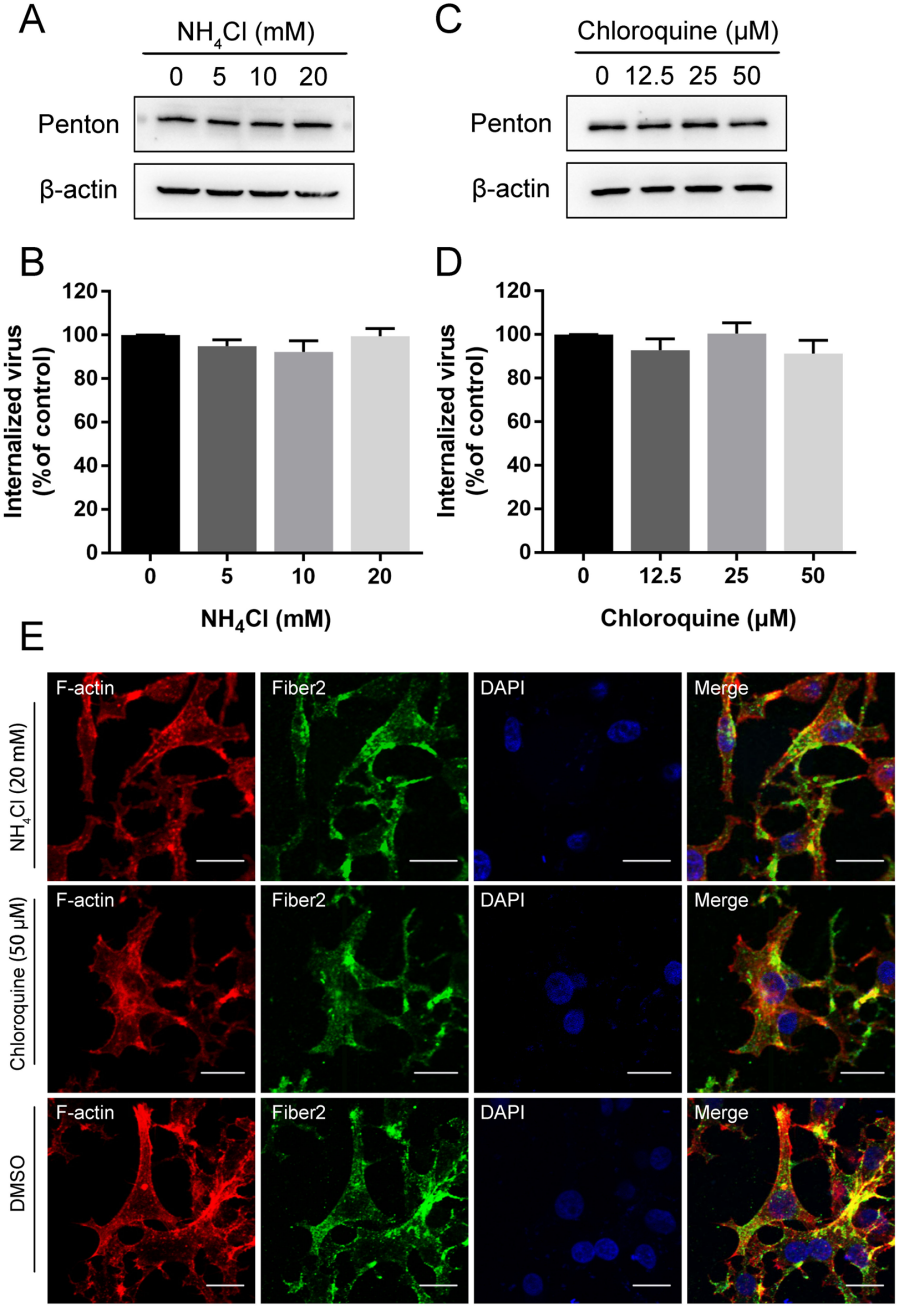
**
FAdV-4 entry is pH-independent. A** Western blot and qPCR analysis after the entry of FAdV-4 into NH_4_Cl-treated cells. **B** Western blot and qPCR analysis after the entry of FAdV-4 into chloroquine-treated cells. **C** CLSM analysis of F-actin (red), anti-FAdV-4 fibre 2 (green) and DAPI (blue) in FAdV-4-infected LMH cells pretreated with NH_4_Cl or chloroquine. Bars, 20 μm. *, *P* < 0.05; **, *P* < 0.01.

To assess whether FAdV-4 entry is pH dependent, we evaluated the effects of two lysosomotropic agents, chloroquine and NH_4_Cl, on the FAdV-4 infection rate of LMH cells. Possible drug-induced cytotoxic effects were assessed by CCK-8 cell viability assay. As shown in Figures 1G and H, respectively, cells tolerated as much as 20 mM NH_4_Cl and 50 µM chloroquine. LMH cells were pretreated with various concentrations of these inhibitors, and then, the cells were inoculated with FAdV-4. Unexpectedly, neither NH_4_Cl (Figures 7A and B) nor chloroquine (Figures 7C and D) led to the inhibition of FAdV-4 internalization. Less pronounced changes were detected in the level of penton protein and the number of viral DNA copies among different samples. Moreover, neither 20 mM NH_4_Cl nor 50 µM chloroquine treatment induced the emission of a green fluorescence signal specific to FAdV-4 fiber2 proteins on the cell membrane, in contrast to their effect on the control cells (Figure 7E). Taken together, these data demonstrate that FAdV-4 entry into LMH cells is pH-independent.

## Discussion

Since July 2015, numerous outbreaks of HHS have occurred in commercial chicken farms in many provinces in China, causing substantial economic losses to the poultry industry [5]. FAdV-4 is the predominant causative agent of HHS. Despite the economic importance of FAdV-4, little is known about its pathogenesis. This HHS agent shows a high affinity for hepatic cells [35]. The LMH chicken hepatocellular carcinoma epithelial cell line, established by Japanese researchers in 1981 [36], has been used as a homologous cell line for the study of host gene expression for years [37]. In addition, LMH cells have been used to isolate and propagate several avian viruses, such as infectious laryngotracheitis virus (ILTV) [38], chicken astroviruses (CAstV) [39], and FAdV-4 [40]. The early steps of the w cycle, namely, binding and entry into the host cell, are crucial determinants of infectivity and are necessary for studying the pathogenic mechanisms of a virus. Previous studies by our team have demonstrated that FAdV-4 uptake into LMH cells occurs within 1 h of exposure, which is considered the optimal time point to measure viral entry. We also showed that proteinase K effectively removes the virus attached to the cell membrane [24]. Thus, in this study, the effect of inhibitors on viral internalization was measured by protease treatment and internalization assay 1 h after FAdV-4 infection at 37 °C. Our results showed that FAdV-4 enters LMH cells in a dynamin- and cholesterol-dependent manner via clathrin-mediated endocytosis accompanied by the involvement of macropinocytosis.

AdVs initially bind to the primary receptor CAR and engage αvβ3 or αvβ5 integrin through the RGD domain in the penton base, triggering signals that induce AdV entry into a cell via receptor-mediated endocytosis [18]. Typically, the internalization of AdV follows the classical clathrin-dependent pathway, delivering the virus to endosomes [41]. However, caveolae-mediated endocytosis [22] and nonselective macropinocytosis [20] have also been shown to serve as alternative routes for AdV internalization. Differences in cell type, virus serotype, and available receptors may account for the different endocytic pathways leveraged by viruses. Indeed, many nonencapsulated viruses have been observed to exploit different endocytosis pathways for entry; these viruses include reovirus [16], rotavirus [42], and parvovirus [43]. Both previous reports and present study [23] showed that clathrin-mediated endocytosis is a major endocytic pathway for AdV entry into cells, which prompts us to hypothesize that FAdV-4 entry is clathrin dependent. As expected, disrupting the assembly of clathrin lattices and preventing the assembly of CCPs decreased FAdV-4 entry, indicating that FAdV-4 enters LMH cells through clathrin-mediated endocytosis. Dynamin, a member of the GTPases and a dynamin-like protein (DLP), plays a critical role in endocytic membrane fission events [26] and is engaged in clathrin- and caveolae-mediated endocytosis but not in macropinocytosis [44]. There is evidence suggesting that dynamin is needed for the internalization of HAdV2 [19], HAdV5 [41], HAdV37 [21], and HAdV-26 [14]. However, little is known about the participation of dynamin in FAdV entry. In the present study, the essential role of dynamin in FAdV-4 entry was demonstrated. This finding also confirmed the important role of dynamin in clathrin-dependent endocytosis. In addition to the involvement of dynamin, clathrin-mediated endocytosis of many types of viruses depends on cell pH; these viruses require the acidic pH in the endosome for uncoating and/or escape from endosomes [30]. Intriguingly, pretreatment of LMH cells with two endosomal acidification inhibitors did not affect FAdV-4 internalization, indicating that FAdV-4entry is pH independent. Upon reaching endosomes, AdVs are thought to gain access to the cytosol by penetrating the endosome membrane, and protein VI in AdVs is critical for the disruption of membrane, which is mediated via its N-terminal amphipathic helix [45]. Notably, the process of releasing protein VI from AdV requires significant viral disassembly within endosomes, which may be triggered by the low pH of these compartments [46]. Hence, the role of endosomal acidification in the endosome escape of FAdV-4 may be a direction for further investigation.

Cholesterol is necessary for most cellular internalization routes, and its depletion affects the formation of plasma membrane structures involved in cargo internalization, including both caveolae and CCPs [28]. An increasing number of viruses have been shown to enter cells via the cholesterol-dependent clathrin-mediated endocytosis pathway [17, 29, 30]. In this study, cholesterol depletion by the pretreatment of cells with MβCD inhibited the entry of FAdV4, which suggested the requirement of cholesterol in FAdV-4 internalization. Since membrane cholesterol also plays an important role in the caveolae-mediated endocytosis pathway, we examined whether FAdV-4 followed this pathway to enter LMH cells. Caveolins are structurally important proteins for the formation and stability of caveolae [28]. Our data showed that the reduction in caveolin level and blockade of the signalling cascade initiated by the caveolae-mediated endocytic pathway did not affect FAdV-4 internalization. Therefore, we conclude that FAdV-4 entry into LMH cells does not involve caveolae-dependent endocytosis.

Macropinocytosis is a receptor- and coat-independent form of endocytosis that is a direct route for efficient entry and infection by some viruses [47–49]. In the present study, when the macropinocytosis pathway was blocked with EIPA, the efficiency of FAdV-4 infection significantly decreased. PI3K and its effectors are critical for the formation of lipid microdomains in ruffles and macropinocytic cups, and therefore, some viruses require the PI3K-AKT pathway for entering cells via macropinocytosis [50, 51]. In this work, we utilized wortmannin, an inhibitor of PI3K, to validate the findings presented above, and the data showed that blocking PI3K activity inhibited FAdV-4 entry. In addition to PI3K, small GTPases such as Rac1 and Rab5 and kinases such as PAK1, PKC, and c-Src are also involved in the macropinocytosis pathway [34]; however, whether these proteins are involved in the regulation of FAdV-4-induced macropinocytosis and the precise mechanism of FAdV-4 entry via this pathway remain to be elucidated.

In conclusion, our study demonstrated for the first time that the entry of FAdV-4 into LMH cells involves a dynamin- and cholesterol-dependent mechanism involving clathrin-mediated endocytosis accompanied by the involvement of macropinocytosis and requires PI3K but not caveolae. The molecular mechanism identified in this study broadens our understanding of the pathways required for FAdV-4 infection and facilitates new strategies for combating HHS.

## Data Availability

The datasets analysed in this study are available from the corresponding authors upon reasonable request.
